# Study on the Quality Component Differences Among Tea Cultivars in Northern Guizhou

**DOI:** 10.3390/molecules31183216

**Published:** 2026-09-11

**Authors:** Manman Gao, Kai Zhang, Huan Wang, Heng Sun

**Affiliations:** 1 School of Public Health, Zunyi Medical University, Zunyi 563000, China; gmm1212@zmu.edu.cn (M.G.);; 2 School of Graduate, Zunyi Medical University, Zunyi 563000, China

**Keywords:** tea cultivars, quality components, volatile aromatic compounds, rOAV

## Abstract

To elucidate how quality is reshaped in introduced tea varieties under Guizhou’s unique ecological conditions and to identify quality advantages and flavor differences between exotic and local germplasm resources, this study investigated the quality characteristics of tea plant germplasm from different geographic origins under Guizhou’s ecological conditions. Quality components and volatile aroma compounds were analyzed in four introduced cultivars (CC4H, SLX, SMCY, HJC1) and two local cultivars (QM601, GZSC). Sensory evaluation indicated that CC4H, SMCY, and HJC1 were more suitable for manufacturing premium green tea in the local area. GC-MS identified 147 volatile compounds, with alcohols present at the highest concentrations (1090.69–1499.18 μg/kg). SMCY had the highest total alcohol content. rOAV analysis confirmed that benzaldehyde, (E)-β-damascone, β-ionone, and 1-octene-3-one are the key contributors to the aroma profile. Among the introduced cultivars, SMCY stands out for its floral and aromatic compounds; SLX is notably rich in floral and fruity aldehydes and esters; CC4H has a distinct roasted sweetness, and HJC1 offers the best freshness. The local variety QM601 exhibits synergistic accumulation of tea polyphenols and fruity terpenes, while GZSC maintains its traditional advantage in the balance of phenolic and amino compounds. These findings provide a theoretical basis for the differentiated deployment of tea cultivars in Guizhou’s tea-growing regions.

## 1. Introduction

The tea plant (*Camellia sinensis* L. O. Kuntze) is a perennial cash crop known for its unique quality characteristics, which arise from the interaction between its genetic background and the cultivation environment [1]. One key factor is the germplasm genotype, which influences the abundance and composition of secondary metabolites. This, in turn, directly impacts the plant’s ability to produce essential flavors such as amino acids, tea polyphenols, and catechins [2]. Regional ecological factors—such as altitude, light intensity, temperature, humidity, and soil physicochemical characteristics—play a crucial role in modulating the metabolic rhythms of tea plants. These factors influence the proportional accumulation of metabolites, ultimately resulting in diverse sensory flavor profiles across different cultivars and production areas [3,4,5,6]. This interaction between genotype and environment suggests that when geographically distinct cultivars are introduced to a common ecological region, the degree of divergence in quality components is determined by the intrinsic metabolic capacity of the cultivars. Additionally, this divergence is shaped by the adaptation of their existing metabolic pathways in response to the new environmental conditions.

As a prime example of high-altitude, premium tea-growing regions in southwest China, Guizhou’s unique natural environment—characterized by “high altitude, limited sunlight, frequent fog, and a pristine ecosystem”—endows its tea with quality advantages such as high amino acid content, a balanced phenolic-to-amino acid ratio, and a delicate yet rich aroma [7]. The abundant local tea plant genetic resources within the province provide the genetic foundation for the high-quality development and differentiated competitiveness of Guizhou’s tea industry. To broaden the genetic diversity of tea plant resources, optimize product flavor profiles, and meet diverse market demands, a large number of high-quality tea plant germplasms from outside the province have been introduced to Guizhou in recent years for local cultivation and domestication. However, after any plant cultivar is introduced into a new environment, its physiological metabolism and quality traits must undergo long-term adaptive remodeling in response to specific ecological factors [8,9]. To date, systematic comparative research on the quality domestication characteristics of tea plant germplasm from different geographical origins under typical ecological conditions in Guizhou remains lacking. In addition, the mechanisms underlying the formation of quality differences between these germplasm sources and Guizhou’s native superior germplasm remain largely unexplored.

In this study, four tea cultivars sourced from different provinces outside Guizhou were examined, with two major tea cultivars grown locally in Guizhou used as controls. Under strictly standardized cultivation management and field conditions, key quality indicators—including free amino acids, tea polyphenols, water-soluble extracts, and volatile aromatic compounds—were systematically measured in each tea cultivar. Furthermore, by combining multivariate statistical analysis and difference analysis methods, a thorough analysis of the phenotypic quality differences among the germplasm sources was conducted. The study aims to reveal the characteristics of quality reshaping in exogenous tea varieties following domestication and adaptation to Guizhou’s unique ecological conditions, and to identify the key points of quality advantages and flavor differences between exogenous and local germplasm. The findings will provide a solid theoretical basis and practical guidance for the innovative utilization of tea germplasm resources in Guizhou’s tea-growing regions, the optimization of high-quality and efficient cultivation techniques, and the targeted development of distinctive flavored tea products.

## 2. Results and Discussion

### 2.1. Analysis of Major Quality Components in Different Tea Cultivars

**Figure 1:**
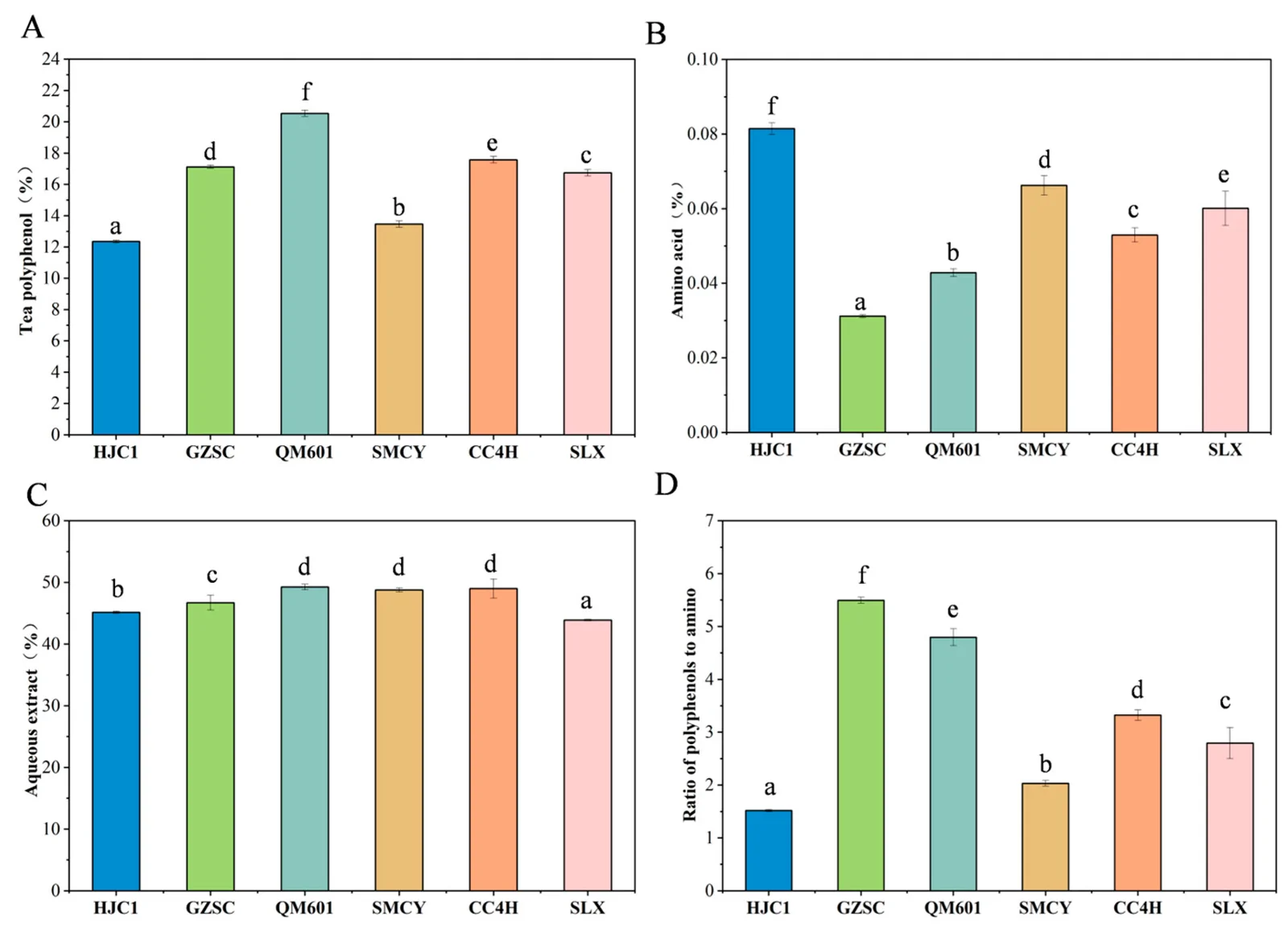
Content of major quality components in different tea cultivars ((**A**): Tea polyphenols; (**B**): Amino acids; (**C**): Aqueous extract; (**D**): Ratio of polyphenols to amino acids). Data represent mean ± SD, and means with the same letter are not significantly different from each other at *p* < 0.05.

The physicochemical factors that determine tea quality grade primarily include key components such as tea polyphenols, free amino acids, and water-soluble extracts [10]. Figure 1 illustrates the distribution characteristics of the major biochemical components in tea samples prepared from the six test cultivars. As a key component of total soluble solids, tea polyphenols are not only the primary substances responsible for the astringent and bitter flavors of tea but also play a significant role in enhancing the richness and body of the tea infusion [11]. In this study, the six tea cultivars showed significant inter-cultivar differences in tea polyphenol content, ranked from highest to lowest as follows: QM601 > CC4H > GZSC > SLX > SMCY > HJC1 (Figure 1A). Differences between any two groups were statistically significant, indicating that QM601 has a distinct cultivar advantage in polyphenol accumulation. The pronounced inter-cultivar differences in tea polyphenol content observed here are consistent with the large natural variation in polyphenol accumulation reported across tea cultivars [12], supporting the conclusion that polyphenol accumulation is strongly genotype-dependent.

Free amino acids are key flavor components that determine the fresh, crisp taste of tea liquor [13]. Experimental data show that the amino acid content of HJC1 is significantly higher than that of the other test cultivars (Figure 1B), suggesting that it has superior potential for a fresh and crisp taste. HJC1’s high free amino acid content suggests its potential as a superior raw material for fresh, umami green tea.

Previous studies have shown that the concentration of aqueous extract directly affects the richness and fullness of the tea’s flavor and is significantly correlated with aroma intensity and the depth of the brewed tea’s color [14]. In this experiment, the water-soluble extract content of QM601, SMCY, and CC4H was higher than that of the other three cultivars (Figure 1C), indicating that these three cultivars have a better chemical basis for shaping the overall taste of the tea infusion.

In addition, the ratio of polyphenols to amino acids (the phenol-to-amino acid ratio) is widely recognized as a key quantitative indicator for evaluating the freshness and balance between bitterness and astringency in green tea; it is generally believed that cultivars with a phenol-to-amino acid ratio below 8 are suitable for producing high-quality green tea [15,16]. As shown in Figure 1D, the phenol-to-amino acid ratios of all six test cultivars were below 8, meeting the basic threshold for green tea production. Other studies have also used the phenol-to-amino acid ratio to evaluate the processing suitability of tea cultivars. Consistent with our results, significant natural variation in the tea polyphenol/free amino acid (TP/FAA) ratio was observed among 87 elite tea cultivars [12], and a proper phenol-to-amino acid ratio was associated with good green tea quality in the Sichuan cultivar CH-1 [17]. Among them, GZSC (5.50) has a relatively high phenol-to-amine ratio; this variety is typically processed into black tea in Yunnan.

### 2.2. Sensory Evaluation Among Different Tea Cultivars

**Figure 2:**
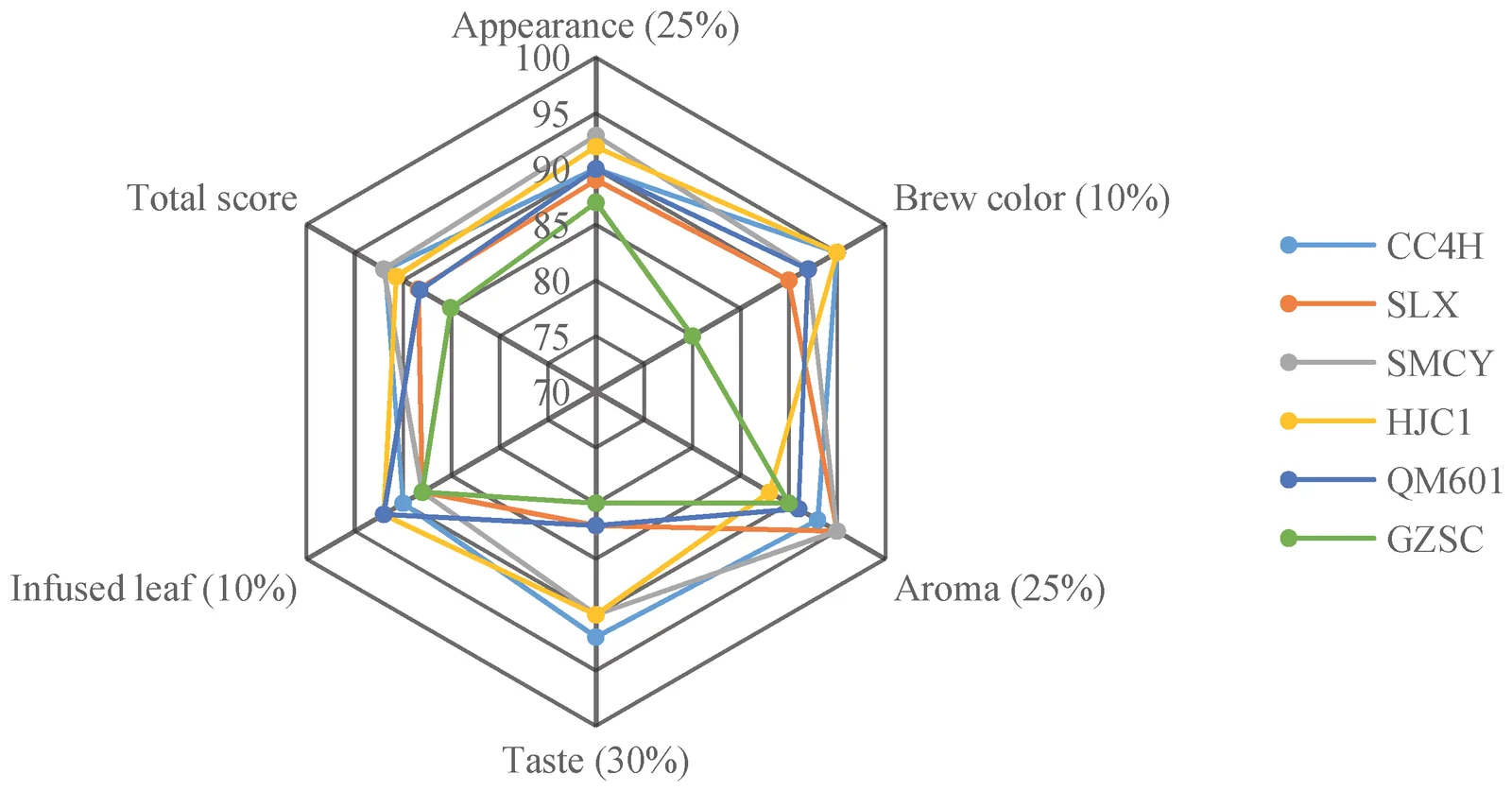
Sensory evaluation of six green teas.

Independent sensory evaluation by 5 professional tea tasters revealed differences in dry-tea appearance, aroma, taste, liquor color, and infused leaves across the six green tea cultivars (Figure 2).

In dry-tea appearance, SMCY exhibited a tender green and glossy appearance with the highest score of 93 points, followed by HJC1, CC4H, QM601, and SLX. GZSC scored only 87 points because of its dull color and irregular shape. In brew color evaluation, CC4H and HJC1 exhibited a tender-green and bright appearance and scored 95 points, followed by SMCY, QM601 and SLX. GZSC showed a distinctive light-purple liquor and obtained a score of 80 points. In aroma evaluation, SMCY and SLX achieved the highest score (95) because of their sweet, long-lasting fragrance. CC4H ranked second with a high and relatively persistent aroma (93). QM601 had a tender fragrance and a score of 91; GZSC possessed a floral note (90); HJC1 had a fresh fragrance and a score of 88. For taste quality, CC4H exhibited the highest score of fresh and mellow (92), followed by SMCY and HJC1 (90). SLX and QM601 showed astringent taste and scored 82 points; GZSC (80) showed bitter-astringent taste.

Based on the weighted evaluation system: appearance of dry tea (25%), brew color (10%), aroma (25%), taste (30%), and infused leaf (10%), the comprehensive sensory scores of the six green teas in descending order were as follows: CC4H (92) = SMCY (92) > HJC1 (91) > SLX (88) = QM601 (88) > GZSC (85). The sensory evaluation results indicated that CC4H, SMCY, and HJC1 were more suitable for manufacturing premium green tea in the local area.

### 2.3. Analysis of Volatile Aromatic Compounds in Different Tea Cultivars

To further investigate the differences in the composition of volatile compounds among different tea cultivars, this study employed headspace microextraction-gas chromatography-mass spectrometry (HS-SPME/GC-MS) to perform qualitative and quantitative analyses of volatile metabolites in six tea cultivars.

**Figure 3:**
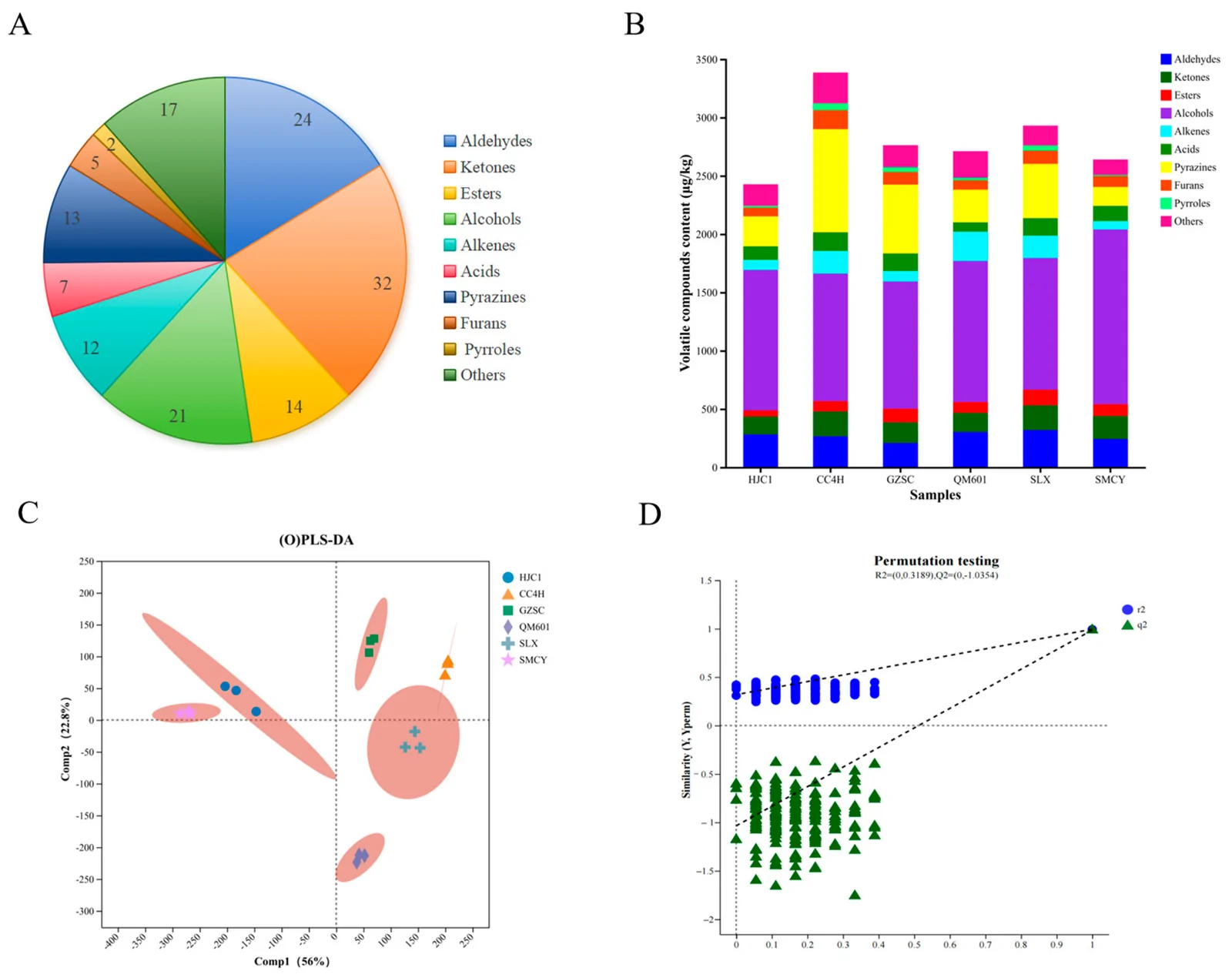
Analysis of volatile compounds in different tea cultivars ((**A**): Pie chart of volatile compound types; (**B**): Stacked histogram of volatile compounds; (**C**): OPLS-DA plot of volatile compounds; (**D**): Permutation test).

A total of 147 volatile compounds were identified and quantified, including 32 ketones, 24 aldehydes, 21 alcohols, 14 esters, 12 alkenes, 13 pyrazines, 7 acids, 5 furans, 2 pyrroles, and 17 other compounds (Figure 3A, Table 1).

Alcohols with floral and sweet notes were the most prevalent substances in teas, which occur not only in free forms but also in the form of glycosides [18]. Among the 147 volatile compounds, alcohols accounted for a relatively high proportion of the total, primarily phenethyl alcohol, benzyl alcohol, geraniol, linalool, and α-pinene, consistent with previous studies on the composition of tea aroma compounds [19]. The dominance of alcohols, particularly linalool, geraniol, and phenethyl alcohol, is consistent with comparative studies on green tea cultivars in which linalool, geraniol, and nonanal were among the most abundant volatiles [20]. Alcohols and hydrocarbons have also been reported as the two most prevalent volatile classes in green tea [21]. The total content of the six types of alcohol compounds showed a clear variety-dependent pattern, ranging from highest to lowest as follows: SMCY (1499.18 μg/kg) > QM601 (1209.36 μg/kg) > HJC (1203.54 μg/kg) > SLX (1130.01 μg/kg) > CC4H (1093.54 μg/kg) > GZSC (1090.69 μg/kg). The extremely high alcohol content in SMCY is a key factor contributing to its excellent aromatic quality. Floral-scented tea cultivars have been reported to accumulate higher levels of linalool and geraniol [22], which may explain the pronounced floral character of SMCY. This makes it a highly promising candidate variety for green tea with distinctive floral flavors. As shown in Table 1, phenethyl alcohol, benzyl alcohol, and linalool were present at high levels in SMCY, QM601, SLX, and CC4H, whereas HJC and GZSC are characterized by phenethyl alcohol, geraniol, and linalool as their primary aromatic components. Alcohols are the primary contributors to the delicate floral and fruity aromas of green tea. Existing research has confirmed that linalool works synergistically to create floral, fruity, and woody notes [23,24]. Geraniol combines fruity, floral, and sweet aromas with a fresh, green leafy note and is a key contributor to green tea’s floral notes, fresh, crisp aroma, and chestnut-like fragrance [25,26]. Phenethyl alcohol primarily contributes sweet and rose-like notes [27,28]. Differences in alcohol group composition among tea cultivars account for the distinct aromatic profiles observed in tea quality.

Among the 24 aldehydes detected, benzaldehyde, nonanal, tetradecanal, and β-cyclocitral were present in relatively high concentrations (Table 1). These components are commonly identified in green tea [29,30] and jointly contribute to tea aroma. A comparison of aldehyde content showed that the total aldehyde content in both SLX and QM601 was higher than in the other four cultivars, particularly benzaldehyde. Tetradecanal content in SLX was significantly higher than in the other five cultivars. Relevant studies have verified that benzaldehyde features a distinct almond odor, while nonanal delivers rose-like notes at low concentrations [31]. Tetradecanal contributes floral and citrus aromas, and β-cyclocitral imparts a minty scent [32]. Collectively, these compounds are vital contributors to tea aroma.

Among the 32 ketone compounds identified, jasmone, β-ionone, 2,3-octanedione, and 6-methylhept-5-en-2-one were present in relatively high concentrations. Among them, jasmone content in SMCY was significantly higher than in other cultivars. Previous studies have indicated that jasmone has floral and creamy-sweet aromas and may contribute to the sweet, mellow floral aroma in green tea [33]. The content of 2,3-octanedione (with notes of grass and fennel) in SLX was significantly higher than in other cultivars. In comparison, the content of 6-methyl-5-hepten-2-one (with notes of apple and mushrooms) in CC4H was significantly higher than in other cultivars [34,35], indicating differentiated flux distribution in the lipoxygenase metabolic pathway among specific cultivars.

A total of 14 ester compounds were identified, with methyl salicylate displaying the highest concentration. Notably, its concentration in SLX was significantly greater than in other cultivars. Methyl salicylate is known for its characteristic minty aroma and is a common ester compound found in green tea [36]. Additionally, the content of methyl palmitate in SMCY was significantly higher than in the other four cultivars. This compound is primarily associated with waxy, oily, and iris-like aromas [37].

The Maillard reaction occurs readily during the high-temperature processing of green tea, yellow tea, oolong tea, and black tea and can produce various aromatic compounds such as pyrazines, furans, pyrroles, and their derivatives [38,39,40]. A total of 13 pyrazines, 5 furans, and 2 pyrroles were identified in this experiment, and the pyrazine and pyrrole contents in CC4H were significantly higher than those in other cultivars (Figure 3). Among these, 2,3-dimethylpyrazine and 2-methylpyrazine are important volatile compounds responsible for the “sweet” and “chestnut-like” characteristics [41]. The pyrazine/pyrrole-enriched profile of CC4H indicates its suitability for producing chestnut- and roasted-aroma green tea.

In terms of olefin compounds, the total content of QM601, CC4H, and SLX was significantly higher than that of the other three cultivars. Among these, the QM601 variety contained significantly higher levels of limonene, isopinoene, γ-terpinene, (3E)-β-basilene, α-terpinene, β-pinene, and cubene than the other cultivars. Studies have confirmed that limonene, isopinoene, γ-terpinene, and cubene all exhibit lemon and citrus fruit aromas [42,43,44], while (3E)-β-ocimene possesses woody and sweet characteristics [45]. The synergistic accumulation of these compounds in QM601 may significantly enhance the release of its floral and fruity notes. In addition, SLX has significantly higher levels of caramene, α-bisabolene, and α-corene than other cultivars, giving it a unique aromatic undertone. Furthermore, the co-occurrence of multiple fruity terpenes in QM601 may endow it with a distinctive fruity-floral character, complementing its polyphenol-rich taste profile.

To visually illustrate the inter-varietal differences in volatile aroma compounds among the six tea cultivars under study (HJC1, CC4H, GZSC, QM601, SLX, and SMCY), this study established an orthogonal partial least squares discriminant analysis (OPLS-DA) model. As shown in Figure 3C, the first principal component (Comp 1) and the second principal component (Comp 2) in the OPLS-DA score plot explain 56.0% and 22.8% of the variance, respectively, with a cumulative variance explained of 78.8%, indicating that the first two principal components effectively capture the main variability in the original data. Based on the sample distribution, the six cultivars exhibit a distinct variety-specific clustering pattern on the scoring plot. Specifically, HJC1 and CC4H are concentrated on the positive end of Comp 1. At the same time, GZSC and QM601 show a high degree of spatial overlap on the negative end of Comp 1, suggesting a certain degree of similarity in their comprehensive metabolic profiles. SMCY and SLX exhibited relatively broad distributions at the positive and negative ends of Comp 2, respectively, reflecting their distinct flavor profiles. Overall, although the cultivars overlap somewhat on the OPLS-DA score plot, each variety forms a relatively distinct cluster, indicating that the comprehensive spectral characteristics of their volatile aromas and biochemical components are variety-specific.

To further validate the effectiveness and reliability of the OPLS-DA model, this study conducted 200 permutation tests (Figure 3D). The results show that in the replacement test, both the original values of R^2^ (the model’s explanatory power for the X variable) and Q^2^ (the model’s predictive ability) were higher than the corresponding values for all replacement random models. Furthermore, the intercept of the Q^2^ regression line was negative, indicating that the model does not exhibit overfitting and possesses good predictive ability and statistical reliability. The above results confirm that the OPLS-DA model based on volatile aroma compounds can effectively distinguish tea plant cultivars from different sources.

In summary, these six tea cultivars differ significantly in their aromatic characteristics, primarily due to variations in aromatic compound content and the proportions of key aromatic compounds.

### 2.4. Confirmation of Odor-Active Compounds in Six Tea Cultivars

A combination of numerous volatile compounds determines the final aroma profile of tea, but not all identified volatile substances make a substantial contribution to the final aroma quality. Odor researchers note that odor compounds with an rOAV (Relative Odor Activity Value) greater than 1 generally contribute to the overall aroma profile of the sample being analyzed. In addition, odor compounds with an rOAV greater than 100 are considered to play a significant role in shaping the overall aroma of the sample being analyzed [46]. Based on this, this experiment calculated the rOAV of each volatile compound to further identify the key flavor-forming compounds in six different tea cultivars.

**Figure 4:**
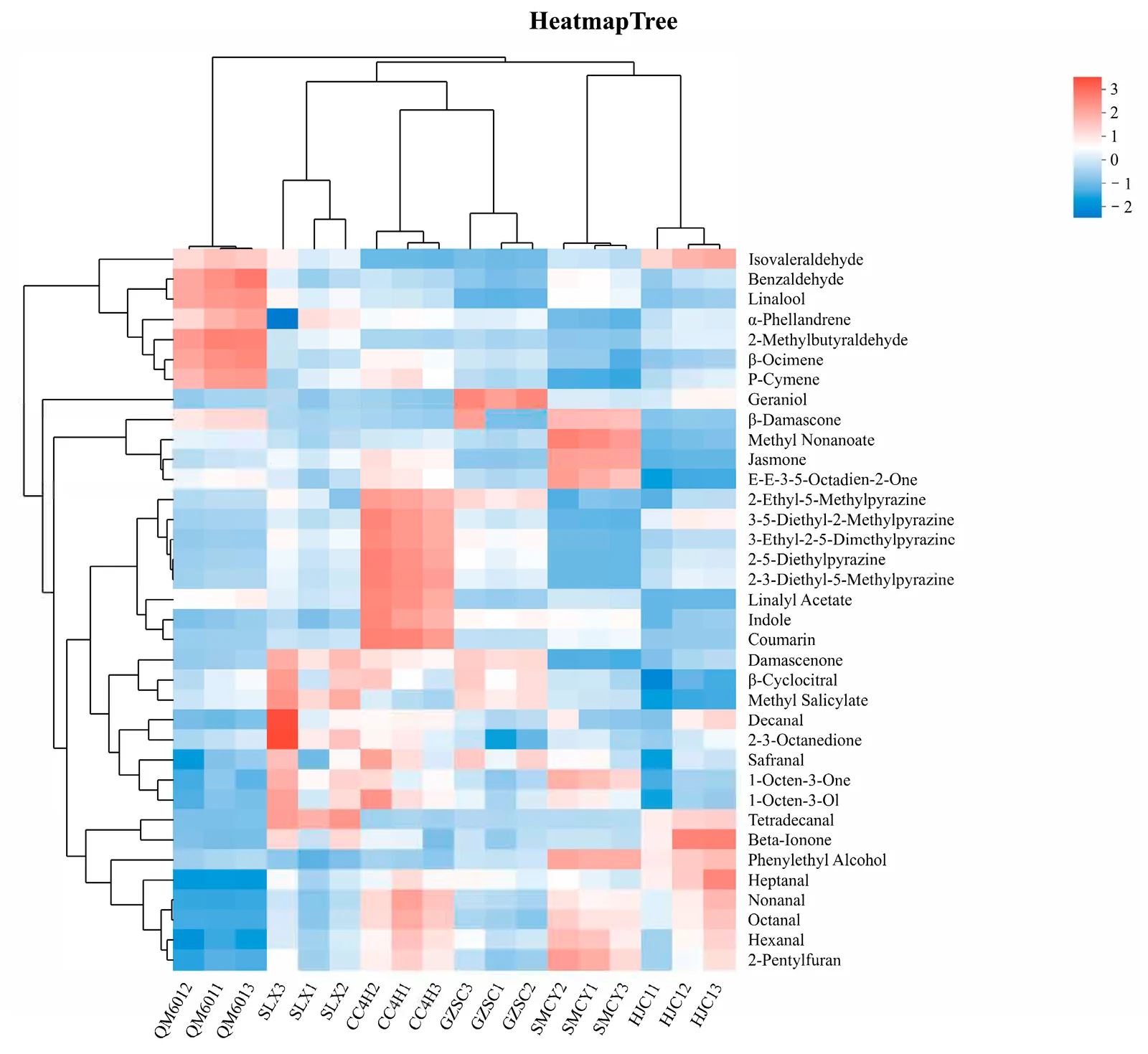
Heat map of potential key aroma compounds in processed teas from 6 different tea cultivars.

As shown in Figure 4 and Table 2, a total of 36 key aroma-active compounds with rOAV values greater than 1 were identified, including aldehydes (11), ketones (7), esters (3), alcohols (4), alkenes (2), pyrazines (5), furans (1), and other classes (3), most of which present floral, fruity, and woody sensory notes. Among these, compounds with higher rOAV values include benzaldehyde (2804.17–6986.11), (E)-β-damascenone (616.33–5253.33), (3E)-β-ocimene (417.17–2018.33), β-ionone (836.51–1315.87), and 1-octene-3-one (531.11–998.89). These compounds were detected in all tea samples tested, and their rOAV values were well above 100, indicating that they form the core framework of the overall aromatic matrix in all samples. On the other hand, heterocyclic compounds with roasted or nutty aromas, such as 3-ethyl-2,5-dimethylpyrazine and 2,3-diethyl-5-methylpyrazine, were also identified as key contributors (all with rOAV values greater than 1), suggesting that heterocyclic compounds formed during thermal processes such as fixation and drying are crucial for the rich roasted and nutty aromas of green tea [47]. Furthermore, linalool, as a key aroma marker for many tea cultivars, had an rOAV greater than 1 in all six test cultivars in this study. Moreover, the rOAV for all cultivars except GZSC exceeded 10, further confirming linalool’s important role in shaping the floral aroma quality of tea [37].

### 2.5. Analysis of the Potential Regulatory Relationship Between Amino Acids and Tea Polyphenols on the Accumulation of Key Volatile Aromatic Compounds

**Figure 5:**
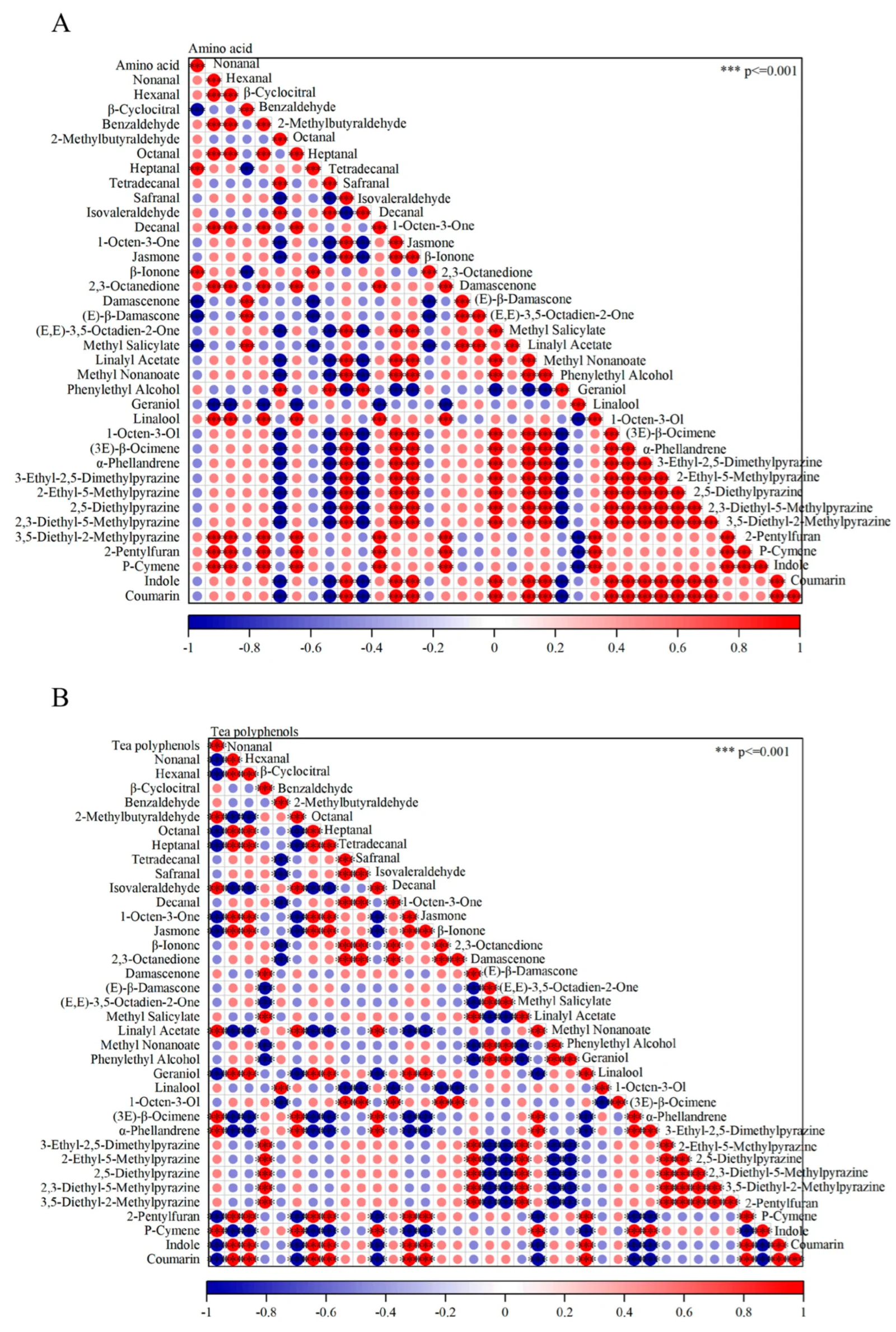
(**A**) Pearson correlation analysis between amino acids and key aroma compounds. (**B**) Pearson correlation analysis between tea polyphenols and key aroma compounds.

To investigate whether the two major non-volatile flavor compounds in tea (amino acids and tea polyphenols) exhibit a statistically significant synergistic or antagonistic relationship with the metabolic accumulation of volatile aroma compounds, this study conducted Pearson correlation analyses between each of these two compounds and key aroma compounds with rOAV values greater than 1 (Figure 5). Given the complex substrate competition and enzymatic cascade reactions within the aroma metabolism network, statistical correlations suggest potential metabolic associations rather than direct causal relationships.

Amino acid content showed a significant negative correlation with β-cyclocitral, damascone, (E)-β-damascone, and methyl salicylate. The underlying mechanism is hypothesized to involve high amino acid concentrations competing with the carotenoid degradation pathway and the phenylpropanoid pathway for shared metabolic precursors or energy carriers, thereby reducing the enzymatic production efficiency of these floral-fruity and fresh-scented compounds [48]. In contrast, amino acids showed a significant positive correlation with 2-methylbutanal, heptanal, tetradecanal, isovaleraldehyde, β-violetone, and phenethyl alcohol. This is primarily because most of the aforementioned aldehydes and alcohols are either direct products of the Strecker degradation of amino acids (such as leucine, isoleucine, and phenylalanine) during thermal processing or secondary derivatives catalyzed and promoted by amino acid metabolic intermediates. The more abundant the precursor substrates, the greater the accumulation of the corresponding products [49].

The tea polyphenol content showed a significant negative correlation with nonanal, hexanal, octanal, heptanal, safranal, 1-octen-3-one, jasmone, geraniol, 1-octen-3-ol, 2-pentylfuran, indole, and coumarin. This may be attributed to the non-competitive inhibitory effect of tea polyphenols on lipoxygenase (LOX) and the activity of its downstream cleavage enzymes, combined with the physical adsorption and acid-mediated degradation effects of polyphenolic compounds on aldehydes and ketones, thereby reducing the content of the aforementioned aroma compounds, which are primarily derived from lipid oxidation [50]. Tea polyphenols showed a significant positive correlation with 2-methylbutanal, isovaleraldehyde, linalyl acetate, linalool, (3E)-β-ocimene, α-phellandrene, and p-cymene. On the one hand, quinone intermediates formed by the oxidation of polyphenols may mediate the oxidative decarboxylation of branched-chain aldehydes [51]. On the other hand, the strong reducing properties and antioxidant protection provided by tea polyphenols can maintain the stability of unsaturated double bonds in terpenes and terpenoids, preventing premature oxidative degradation during processing and thereby showing a phenotypic association with the co-accumulation of terpenoid aroma compounds.

## 3. Materials and Methods

### 3.1. Main Chemicals

The main chemicals used in this study were ninhydrin (AR, 98%, Shanghai Haohong Biomedical Technology Co., Ltd., Shanghai, China); Folin–Ciocalteu (biotechnology grade, 1 mol/L, Shanghai Eanchen Chemical Technology Co., Ltd., Shanghai, China); L-theanine (standard products, 98%, Shanghai Eanchen Chemical Technology Co., Ltd.); and tea polyphenol (standard products, >98%, Shanghai Eanchen Chemical Technology Co., Ltd.).

The 20 mL headspace vials with 18 mm magnetic PTFE/silicone caps were acquired from Agilent Technologies Inc. (Palo Alto, CA, USA). The manual holder and divinylbenzene/carbon wide range/polydimethylsiloxane (DVB/CWR/PDMS) fiber assembly for headspace solid-phase microextraction (HS-SPME) were sourced from Supelco (Bellefonte, PA, USA).

### 3.2. Plant Materials

**Figure A1:**
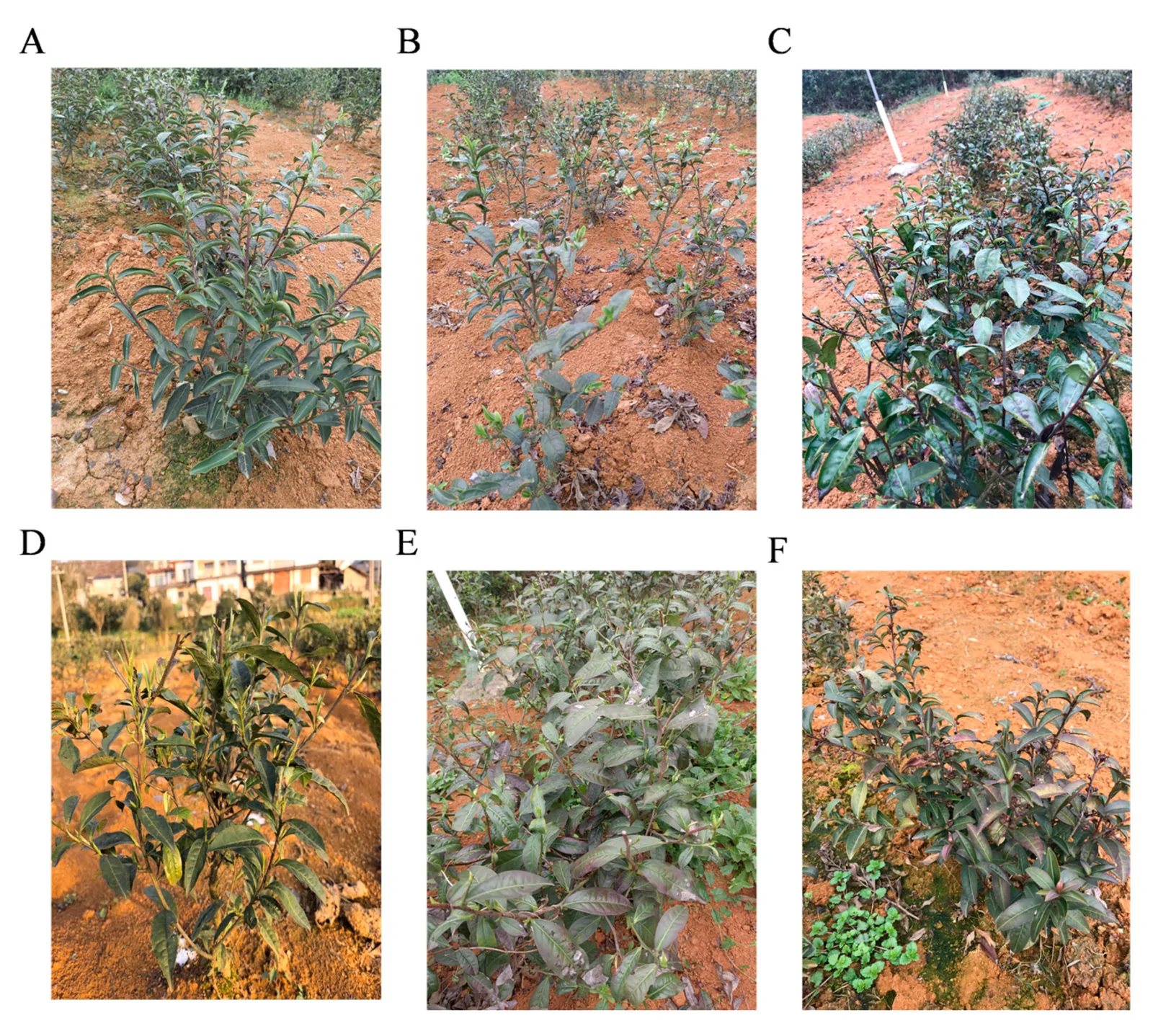
Morphological phenotypes of 6 tea cultivars ((**A**): CC4H, (**B**): SLX, (**C**): SMCY, (**D**): HJC1, (**E**): QM601, (**F**): GZSC).

In this study, six tea cultivars (HJC 1, CC4H, GZSC, QM601, SLX and SMCY) were planted at the Egongba Tea Germplasm Repository, located in Xinglong Town, Meitan County, Zunyi City, Guizhou Province (Figure A1). All tea trees used in this study were 4 years old.

### 3.3. Characterization of Tea Samples

Fresh tender tea shoots (one bud and one leaf) were collected in the early spring before the Qingming Festival. All samples were dried to constant weight in an oven at 103 °C, then pulverized and sieved through a 60-mesh screen for further analysis. The fresh tender tea shoots were processed into green tea using the pan-firing technique [19]. Sensory evaluation of the tea was performed in accordance with GB/T 23776-2018 (Methods for sensory evaluation of tea) [52].

The contents of tea polyphenols were determined by the spectrophotometric method specified in GB/T 8313-2018 (Determination of tea polyphenols and catechins in tea) [53]. The total free amino acid content was measured using the spectrophotometric method described in GB/T 8314-2013 (Tea-Determination of total free amino acids) [54]. The water extract content was determined in accordance with GB/T 8305-2013 (Tea-Determination of water extracts) [55].

The ratio of tea polyphenols to free amino acids (phenol-amino ratio) was calculated as follows: phenol-amino ratio = tea polyphenol content/total free amino acid content. Each experiment was repeated 3 times.

### 3.4. Analysis of Volatile Metabolites in Tea Samples Based on HS-SPME-GC/MS

#### 3.4.1. Headspace Solid-Phase Microextraction Conditions

We accurately weighed 1.0 g of tea powder into a 20 mL headspace vial, then added 2.5 µL of internal standard (n-pentadecane-d32, 98% purity, 50 µg/mL) and 4 mL of saturated sodium chloride aqueous solution. Equilibrate the sample at 80 °C and 500 rpm for 20 min. Then extract using a 120 µm DVB/CWR/PDMS solid-phase microextraction fiber at 80 °C and 500 rpm for 10 min. After extraction, the fiber was thermally desorbed in the GC injection port at 250 °C for 5 min.

#### 3.4.2. Chromatographic and Mass Spectrometry Conditions

A VF-WAXms column (25 m × 0.25 mm × 0.2 µm, Agilent CP9204) was used for the analysis. The injector temperature was set to 240 °C. High-purity helium was utilized as the carrier gas at a flow rate of 1.0 mL/min, along with a septum purge flow of 3 mL/min. The oven temperature was programmed to increase from 40 °C to 120 °C at a rate of 8 °C/min, and then to 230 °C at a rate of 20 °C/min, maintaining this temperature for 4.5 min. The total run time was 20 min. Mass spectrometry was conducted in full-scan mode (*m*/*z* 35–500) using an electron impact (EI) ionization source. The ion source temperature was kept at 250 ◦C, with the electron energy set to 70 eV.

#### 3.4.3. Qualitative and Quantitative Methods for Volatile Compounds

Volatile compounds were identified by comparing the calculated retention indices (RI, determined from n-alkanes C8–C30) with those in the standard mass spectra database (NIST-2023, GC-Orbitrap flavor and fragrances v1.0). The following formula was used to quantify the volatile compounds using the internal standard method.

Content of volatile compound (μg/kg) = S1 × m0/S0 × M

S1, peak area of each compound; S0, peak area of internal standard;

m0, the quality of the internal standard (μg); M, the quality of tea sample (g).

#### 3.4.4. Relative Odor Activity Value

The relative odor activity value (rOAV) was used to evaluate each volatile compound’s contribution to the overall tea aroma. Based on the semi-quantitative concentrations of the volatile compounds, the flavor thresholds of volatile components in references were consulted, and the rOAV of volatile components was calculated according to formula [56]:

rOAV = C/OT

where C is the relative content of volatile compounds, μg/kg; OT is the aroma threshold of volatile compounds, μg/kg.

### 3.5. Statistical Analysis

Data on volatile compounds were analyzed using Excel 2024 software, with results presented as ‘mean ± standard deviation’. A bar chart was created, and Pearson correlation analysis was performed using Origin 2026SR1 software. The heatmap was generated with TBtools-IIv2.323 software, and OPLS-DA analysis was conducted using SIMCA-P 14.1 software.

## 4. Conclusions

In summary, the six tea cultivars from different regions showed strong quality when cultivated under the same ecological conditions in Meitan, Guizhou. Among the introduced cultivars, SMCY showed the richest floral and aromatic profiles; SLX stood out for its high concentration of floral and fruity aldehydes and esters; CC4H displayed notable roasted sweetness; and HJC1 offered the best amino acid-derived freshness. The local variety QM601 exhibits a distinct advantage in the synergistic accumulation of tea polyphenols and fruity terpenes. In contrast, GZSC, with its relatively high phenol-to-amine ratio, may be more suitable for black tea processing. The sensory evaluation results indicated that CC4H, SMCY, and HJC1 were more suitable for manufacturing premium green tea in the local area. On this basis, we recommended that tea-growing regions in Guizhou adopt a flavor-oriented, differentiated deployment of introduced cultivars to develop diverse and distinctive tea products, while also conducting genetic improvement focused on flavor harmony and ecological adaptability using local cultivars as core parental lines.

## Figures and Tables

**Table 1:** The relative content of aroma components in six different tea cultivars (μg/kg).

Compounds	Retention Time	RI	Library RI	CAS	HJC1	CC4H	GZSC	QM601	SLX	SMCY
	Aldehydes									
Nonanal	7.532	1391	1391	124-19-6	28.23 ± 4.86 ab	32.53 ± 2.84 a	20.33 ± 0.80 cd	13.27 ± 0.15 e	19.93 ± 2.30 d	27.67 ± 0.91 b
4-Methylbenzaldehyde	10.82	1619	1623	104-87-0	2.50 ± 0.29 b	2.20 ± 0.17 b	3.76 ± 0.22 a	2.10 ± 0.14 b	2.42 ± 0.33 b	3.21 ± 0.13 ab
Hexanal	3.194	1079	1083	66-25-1	5.44 ± 0.79 bc	5.98 ± 0.38 ab	5.10 ± 0.29 c	3.58 ± 0.22 d	4.93 ± 0.29 c	6.21 ± 0.38 a
Valeraldehyde	2.319	987	979	110-62-3	3.58 ± 0.32 ab	4.08 ± 0.15 a	2.76 ± 0.21 cd	2.54 ± 0.09 d	3.83 ± 0.64 ac	3.51 ± 0.26 bc
β-Cyclocitral	10.783	1615	1611	432-25-7	7.48 ± 0.91 d	11.20 ± 1.31 b	11.77 ± 0.81 ab	10.25 ± 0.64 c	12.06 ± 1.99 a	9.77 ± 0.35 c
Furfural	8.607	1461	1461	98-01-1	1.47 ± 0.15 c	8.49 ± 0.69 a	3.15 ± 0.35 b	2.47 ± 0.09 b	4.11 ± 1.73 b	1.69 ± 0.23 c
Salicylaldehyde	11.418	1676	1670	90-02-8	1.83 ± 0.25 b	2.29 ± 0.12 a	2.05 ± 0.14 ab	1.75 ± 0.12 c	2.60 ± 0.29 a	1.93 ± 0.14 bc
Benzaldehyde	9.458	1518	1520	100-52-7	83.40 ± 10.11 b	85.87 ± 4.50 b	67.30 ± 3.55 c	167.67 ± 11.50 a	85.57 ± 11.95 b	108.30 ± 7.43 b
2,6-Dimethyl-5-Heptenal	6.313	1312	1357	106-72-9	4.41 ± 0.40 c	6.30 ± 0.60 a	5.61 ± 0.42 ab	5.30 ± 0.35 b	6.52 ± 1.06 a	4.53 ± 0.26 c
2-Methylbutyraldehyde	1.933	946	914	96-17-3	17.13 ± 1.67 b	10.13 ± 0.41 d	10.92 ± 1.02 d	46.87 ± 3.02 a	18.23 ± 3.06 b	6.31 ± 0.50 e
5-Methylfurfural	10.216	1571	1570	620-02-0	4.55 ± 0.57 d	28.20 ± 1.97 a	11.77 ± 0.81 b	8.13 ± 0.42 c	12.00 ± 2.13 b	4.32 ± 0.29 d
Octanal	5.933	1287	1289	124-13-0	4.71 ± 0.78 bc	5.48 ± 0.48 b	3.02 ± 0.22 d	2.08 ± 0.03 e	3.38 ± 0.50 cd	5.03 ± 0.27 bc
Methylglyoxal	2.313	986	970	78-98-8	0.30 ± 0.03 ab	0.31 ± 0.03 a	0.23 ± 0.02 c	0.18 ± 0.01 d	0.30 ± 0.04 ab	0.25 ± 0.03 bc
Heptanal	4.439	1183	1185	111-71-7	11.75 ± 2.00 a	9.75 ± 0.90 ab	9.12 ± 0.51 b	4.24 ± 0.05 c	8.13 ± 1.02 b	8.72 ± 0.63 b
Pyrrole-2-Carboxaldehyde	13.935	2029	2030	1003-29-8	6.24 ± 0.77 c	11.73 ± 0.90 a	5.42 ± 0.21 c	4.36 ± 0.29 d	5.40 ± 0.85 c	3.07 ± 0.06 e
2-Heptenal	6.447	1321	1323	18829-55-5	0.43 ± 0.07 d	0.93 ± 0.19 a	0.57 ± 0.08 c	0.53 ± 0.11 c	1.03 ± 0.17 a	0.92 ± 0.03 ab
Tetradecanal	13.325	1925	1930	124-25-4	77.97 ± 9.08 b	23.30 ± 2.16 d	27.73 ± 1.19 d	10.26 ± 0.26 e	109.67 ± 7.77 a	29.57 ± 0.21 c
Safranal	11.062	1642	1648	116-26-7	9.85 ± 1.13 b	11.80 ± 1.20 a	11.70 ± 0.70 a	9.28 ± 0.71 b	11.02 ± 1.53 a	10.93 ± 0.55 a
Isovaleraldehyde	1.951	948	918	590-86-3	5.56 ± 0.63 a	1.11 ± 0.04 d	1.25 ± 0.10 d	5.11 ± 0.31 a	3.41 ± 0.58 c	2.59 ± 0.20 c
2-Hexenal	4.894	1216	1216	6728-26-3	0.55 ± 0.05 c	0.23 ± 0.02 d	0.76 ± 0.05 a	0.37 ± 0.02 d	0.45 ± 0.05 c	1.34 ± 0.07 a
Cis-Cinnamaldehyde	12.942	1866	1884	57194-69-1	0.13 ± 0.01 b	0.17 ± 0.01 a	0.16 ± 0.02 a	0.13 ± 0.01 b	0.18 ± 0.04 a	0.14 ± 0.02 b
Decanal	9.137	1496	1498	112-31-2	2.43 ± 0.62 b	2.61 ± 0.03 ab	2.10 ± 0.13 bc	1.67 ± 0.05 c	3.02 ± 0.99 a	2.09 ± 0.51 bc
Citral	11.906	1729	1732	141-27-5	0.74 ± 0.10 b	0.38 ± 0.04 d	1.31 ± 0.06 a	0.47 ± 0.04 cd	0.42 ± 0.04 d	0.69 ± 0.02 bc
α-Cyclocitral	8.169	1433	1425	432-24-6	0.42 ± 0.05 c	0.63 ± 0.05 a	0.60 ± 0.04 a	0.47 ± 0.02 bc	0.63 ± 0.05 a	0.48 ± 0.04 b
	Ketones									
2,3-Pentanedione	2.968	1055	1054	600-14-6	1.27 ± 0.12 c	2.51 ± 0.23 a	1.76 ± 0.19 b	1.07 ± 0.03 c	2.02 ± 0.39 ab	1.01 ± 0.02 c
1-Octen-3-One	6.116	1300	1301	4312-99-6	1.75 ± 0.25 c	2.52 ± 0.26 b	1.96 ± 0.17 c	1.59 ± 0.18 c	2.84 ± 0.33 a	3.00 ± 0.17 a
3-Penten-2-One	3.697	1124	1122	3102-33-8	3.79 ± 0.64 a	2.23 ± 0.80 bc	1.99 ± 0.27 c	2.93 ± 0.52 ab	2.33 ± 0.02 bc	2.21 ± 0.18 bc
Methyl Ethyl Ketone	1.87	939	945	78-93-3	0.57 ± 0.08 ab	0.41 ± 0.03 c	0.37 ± 0.04 c	0.69 ± 0.06 a	0.56 ± 0.18 ab	0.50 ± 0.05 bc
Jasmone	13.435	1943	1961	488-10-8	3.46 ± 0.51 d	20.20 ± 1.74 a	6.79 ± 0.21 c	11.67 ± 0.97 b	15.03 ± 1.85 a	30.83 ± 0.40 a
Mesityl Oxide	3.767	1130	1127	141-79-7	6.56 ± 0.34 d	13.57 ± 0.75 b	15.03 ± 0.40 a	14.60 ± 1.23 ab	23.03 ± 2.19 a	6.87 ± 0.47 d
3-Octen-2-One	7.715	1403	1396	1669-44-9	1.17 ± 0.17 c	2.51 ± 0.17 a	2.11 ± 0.22 b	1.30 ± 0.05 c	1.99 ± 0.17 b	1.93 ± 0.14 b
Benzophenone	16.254	2500	2508	119-61-9	1.76 ± 0.27 b	2.22 ± 0.33 a	2.05 ± 0.05 ab	1.60 ± 0.06 b	2.05 ± 0.16 ab	1.29 ± 0.03 c
3-Octanone	5.409	1251	1253	106-68-3	1.08 ± 0.14 ab	1.12 ± 0.05 a	1.00 ± 0.10 b	0.71 ± 0.05 c	1.05 ± 0.18 ab	1.21 ± 0.05 a
2-Octanone	5.862	1282	1285	111-13-7	2.62 ± 0.38 c	3.24 ± 0.14 a	2.46 ± 0.19 c	1.55 ± 0.08 d	2.89 ± 0.47 bc	2.84 ± 0.22 bc
2′-Aminoacetophenone	14.968	2230	2223	551-93-9	0.74 ± 0.12 a	0.56 ± 0.03 bc	0.48 ± 0.00 c	0.25 ± 0.01 d	0.52 ± 0.06 c	0.44 ± 0.02 c
Isophorone	10.399	1584	1591	78-59-1	1.51 ± 0.21 b	1.69 ± 0.11 a	1.39 ± 0.09 c	0.88 ± 0.06 d	1.60 ± 0.19 ab	1.13 ± 0.04 c
β-Ionone	13.411	1939	1941	79-77-6	27.63 ± 3.67 a	20.23 ± 2.45 b	19.57 ± 1.02 b	17.57 ± 0.21 b	23.30 ± 2.69 ab	20.23 ± 0.21 b
2-Methyl-1-Penten-3-One	3.034	1062	1069	25044-01-3	6.66 ± 0.83 c	8.39 ± 0.58 a	5.49 ± 0.51 d	7.92 ± 0.36 ab	6.73 ± 1.26 c	5.09 ± 0.24 d
2-Heptanone	4.393	1179	1182	110-43-0	8.88 ± 1.23 ab	9.22 ± 0.47 a	7.83 ± 0.60 c	5.01 ± 0.31 d	8.07 ± 1.27 bc	8.75 ± 0.80 ab
2,3-Octanedione	6.489	1324	1335	585-25-1	27.50 ± 2.72 bc	31.27 ± 2.05 ab	22.73 ± 4.21 c	27.00 ± 1.30 bc	39.00 ± 7.66 a	27.50 ± 1.65 bc
2(5H)-Furanone	12.1	1753	1743	497-23-4	0.17 ± 0.02 c	0.35 ± 0.03 a	0.17 ± 0.03 c	0.08 ± 0.01 d	0.31 ± 0.14 ab	0.26 ± 0.03 b
6-Methyl-5-Hepten-2-One	6.662	1335	1336	110-93-0	35.00 ± 3.38 c	49.90 ± 4.83 a	46.53 ± 3.07 ab	38.30 ± 1.99 c	44.60 ± 7.25 b	44.93 ± 2.27 b
Nerylacetone	12.855	1853	1838	3879-26-3	9.85 ± 1.31 c	15.47 ± 2.04 a	17.07 ± 1.11 a	10.59 ± 0.72 c	14.40 ± 1.90 ab	12.27 ± 0.32 b
1-Hepten-3-One	5.159	1234	1221	2918-13-0	0.29 ± 0.10 b	0.32 ± 0.09 b	0.32 ± 0.04 b	0.38 ± 0.27 b	0.51 ± 0.35 a	0.20 ± 0.01 c
2,4-Dimethyl-3-Hexanone	4.18	1162	1178	18641-70-8	2.44 ± 0.30 c	3.84 ± 0.61 a	3.52 ± 0.21 ab	2.21 ± 0.11 c	4.54 ± 1.54 a	2.16 ± 0.10 c
Damascenone	12.609	1818	1813	23726-93-4	0.32 ± 0.05 d	0.56 ± 0.03 b	0.62 ± 0.02 a	0.31 ± 0.02 d	0.67 ± 0.08 a	0.19 ± 0.01 e
(E)-β-Damascone	12.909	1861	1830	23726-91-2	0.62 ± 0.11 c	1.00 ± 0.13 b	2.25 ± 3.40 ab	4.17 ± 0.26 a	1.27 ± 0.13 bc	5.25 ± 0.12 a
Furaneol	13.947	2031	2031	3658-77-3	2.70 ± 0.36 c	8.67 ± 1.17 a	5.54 ± 0.30 b	2.01 ± 0.53 c	4.73 ± 0.67 b	1.89 ± 0.05 c
(E, E)-3,5-Octadien-2-One	9.424	1516	1522	38284-27-4	3.38 ± 0.36 d	8.61 ± 0.62 a	6.04 ± 0.21 c	7.97 ± 0.43 b	6.14 ± 0.80 c	11.03 ± 0.70 a
1-(3,5-Dimethylpyrazinyl)-Ethanone	11.347	1669	1629	54300-08-2	0.28 ± 0.03 b	0.55 ± 0.04 a	0.34 ± 0.03 b	0.14 ± 0.01 d	0.30 ± 0.03 bc	0.10 ± 0.01 e
2-Pentanone	2.308	986	983	107-87-9	0.07 ± 0.01 b	0.10 ± 0.00 a	0.08 ± 0.01 b	0.07 ± 0.00 b	0.10 ± 0.02 a	0.09 ± 0.01 ab
2,6-Dimethyl-4-Hepten-3-One	12.19	1764	1754	56259-14-4	0.26 ± 0.03 c	0.30 ± 0.02 b	0.25 ± 0.02 c	0.21 ± 0.01 d	0.40 ± 0.05 a	0.34 ± 0.00 b
2,5-Dimethyl-3-Hexanone	4.234	1167	1145	1888-57-9	0.05 ± 0.02 b	0.13 ± 0.04 a	0.09 ± 0.04 ab	0.05 ± 0.00 b	0.11 ± 0.11 ab	0.04 ± 0.01 b
2-Nonanone	7.443	1386	1388	821-55-6	0.52 ± 0.09 c	0.68 ± 0.05 b	0.54 ± 0.06 c	0.30 ± 0.01 d	0.54 ± 0.10 c	2.22 ± 0.19 a
2-Pentadecanone	13.883	2019	2019	2345-28-0	0.27 ± 0.05 c	0.42 ± 0.03 a	0.34 ± 0.03 b	0.24 ± 0.03 c	0.34 ± 0.04 b	0.26 ± 0.00 c
Ethyl Vinyl Ketone	2.629	1020	1019	1629-58-9	0.21 ± 0.03 b	0.11 ± 0.01 c	0.15 ± 0.02 c	0.19 ± 0.02 bc	0.24 ± 0.04 b	0.43 ± 0.03 a
	Esters									
Methyl Palmitate	14.911	2218	2209	112-39-0	4.21 ± 0.48 d	5.13 ± 0.27 c	6.57 ± 0.31 a	5.75 ± 0.06 b	5.29 ± 0.74 c	10.10 ± 0.30 a
Methyl Hexanoate	4.469	1185	1184	106-70-7	6.33 ± 0.69 a	3.19 ± 0.20 c	5.23 ± 0.41 b	2.21 ± 0.09 d	3.78 ± 0.52 c	6.15 ± 0.58 a
(Z)-Acetate 3-Hexen-1-Ol	6.905	1351	1327	3681-71-8	2.83 ± 0.29 a	0.23 ± 0.01 e	1.56 ± 0.07 c	2.42 ± 0.14 b	0.71 ± 0.10 d	0.18 ± 0.01 e
Phenethyl Acetate	12.364	1785	1813	103-45-7	2.05 ± 0.31 b	0.82 ± 0.07 d	0.77 ± 0.03 d	1.05 ± 0.08 c	0.73 ± 0.12 d	3.47 ± 0.23 a
Pentyl Hexanoate	9.331	1509	1510	540-07-8	0.19 ± 0.04 c	0.23 ± 0.02 b	0.16 ± 0.01 d	0.09 ± 0.00 e	0.22 ± 0.02 b	0.14 ± 0.01 d
Methyl Octanoate	7.48	1388	1389	111-11-5	1.10 ± 0.20 c	1.22 ± 0.13 bc	1.04 ± 0.06 c	1.27 ± 0.08 b	1.14 ± 0.16 c	3.00 ± 0.26 a
Methyl Salicylate	12.271	1774	1766	119-36-8	29.87 ± 4.14 d	61.93 ± 6.83 c	93.47 ± 5.03 a	69.93 ± 4.82 c	112.17 ± 14.46 a	65.43 ± 2.06 c
Methyl Anthranilate	15.062	2250	2260	134-20-3	3.03 ± 0.51 c	4.47 ± 0.38 a	1.92 ± 0.04 d	2.63 ± 0.28 c	1.83 ± 0.22 d	2.02 ± 0.09 d
Methyl Linoleate	16.233	2496	2482	112-63-0	1.97 ± 0.21 b	1.94 ± 0.10 b	3.17 ± 0.10 a	1.93 ± 0.15 b	2.29 ± 0.27 b	5.57 ± 0.16 a
Linalyl Acetate	9.445	1517	1555	115-95-7	0.45 ± 0.04 d	6.92 ± 0.59 a	1.41 ± 0.10 c	3.72 ± 0.30 b	2.58 ± 0.29 b	2.40 ± 0.11 b
(Z)-Benzoate 3-Hexen-1-Ol	14.475	2130	2126	25152-85-6	0.05 ± 0.01 a	0.05 ± 0.00 a	0.04 ± 0.00 ab	0.03 ± 0.00 b	0.06 ± 0.01 a	0.05 ± 0.01 a
γ-Caprolactone	11.627	1696	1694	695-06-7	1.44 ± 0.16 c	1.91 ± 0.08 a	1.48 ± 0.07 c	1.18 ± 0.07 d	1.82 ± 0.21 ab	1.89 ± 0.11 ab
γ-Decalactone	14.589	2153	2163	706-14-9	0.33 ± 0.05 c	0.56 ± 0.04 a	0.41 ± 0.02 b	0.23 ± 0.01 d	0.42 ± 0.05 b	0.32 ± 0.02 c
Methyl Nonanoate	9.046	1490	1493	1731-84-6	0.08 ± 0.01 d	0.17 ± 0.01 b	0.14 ± 0.01 c	0.18 ± 0.00 a	0.14 ± 0.02 c	0.39 ± 0.02 a
	Alcohols									
Phenylethyl Alcohol	13.236	1910	1907	60-12-8	681.67 ± 51.48 a	424.67 ± 15.28 c	491.00 ± 8.72 b	444.33 ± 15.53 c	378.67 ± 31.72 d	766.33 ± 10.21 a
Geraniol	12.787	1844	1847	106-24-1	105.20 ± 14.38 a	60.97 ± 5.25 c	179.33 ± 8.08 a	66.57 ± 4.68 c	67.23 ± 8.47 c	88.10 ± 2.51 b
Linalool	9.851	1546	1547	78-70-6	72.63 ± 8.33 c	113.00 ± 6.24 b	42.23 ± 1.99 d	261.33 ± 11.24 a	144.00 ± 19.52 b	147.33 ± 7.23 b
1-Octanol	9.996	1556	1557	111-87-5	32.03 ± 4.73 b	37.47 ± 1.45 a	25.83 ± 1.27 c	20.03 ± 0.93 d	31.43 ± 4.34 b	29.50 ± 1.32 b
1-Undecanol	11.707	1705	1719	112-42-5	2.94 ± 0.47 c	4.48 ± 0.41 a	4.32 ± 0.15 a	3.20 ± 0.25 c	4.73 ± 0.48 a	2.80 ± 0.13 c
1-Octen-3-Ol	8.375	1446	1450	3391-86-4	19.70 ± 2.44 c	29.10 ± 3.46 a	23.33 ± 1.55 b	19.30 ± 0.98 c	27.93 ± 4.35 a	26.30 ± 1.51 ab
Benzyl Alcohol	13.005	1875	1870	100-51-6	195.00 ± 20.95 d	276.00 ± 9.54 c	249.67 ± 6.81 c	204.33 ± 8.96 d	304.00 ± 29.05 b	348.33 ± 10.69 a
1-Penten-3-Ol	4.073	1154	1157	616-25-1	0.51 ± 0.05 d	0.48 ± 0.04 d	0.54 ± 0.03 cd	0.66 ± 0.04 c	0.71 ± 0.13 b	0.94 ± 0.06 a
α-Terpineol	11.576	1691	1688	98-55-5	31.73 ± 4.18 d	57.77 ± 3.84 b	11.77 ± 0.45 e	117.33 ± 8.08 a	56.07 ± 7.23 b	23.40 ± 0.53 c
Epi-Cubenol	14.124	2063	2067	19912-67-5	1.00 ± 0.12 d	4.31 ± 0.48 b	0.92 ± 0.02 d	0.78 ± 0.07 d	7.27 ± 0.73 a	0.87 ± 0.02 d
Hotrienol	10.683	1606	1603	29957-43-5	1.39 ± 0.16 d	18.13 ± 1.42 b	4.82 ± 0.11 c	22.93 ± 1.74 a	15.77 ± 1.86 b	2.96 ± 0.09 c
Nerol	12.451	1796	1797	106-25-2	5.45 ± 0.89 b	1.66 ± 0.17 d	4.01 ± 0.19 c	4.15 ± 0.34 c	1.88 ± 0.31 d	2.02 ± 0.10 d
1-Pentanol	5.307	1244	1250	71-41-0	32.23 ± 2.06 c	41.67 ± 4.66 b	30.77 ± 2.54 c	28.13 ± 1.67 c	56.70 ± 8.41 a	27.33 ± 2.39 c
1-Phenylethanol	12.552	1809	1821	98-85-1	0.89 ± 0.12 d	1.47 ± 0.09 b	2.49 ± 0.09 a	0.94 ± 0.05 d	2.73 ± 0.38 a	5.39 ± 0.61 a
Cis-Nerolidol	13.967	2035	2044	142-50-7	1.56 ± 0.22 d	4.89 ± 0.47 a	2.97 ± 0.23 c	1.41 ± 0.13 d	3.18 ± 0.33 c	2.75 ± 0.09 c
Isogeraniol	12.534	1807	1820	5944-20-7	0.88 ± 0.14 c	1.27 ± 0.10 b	1.32 ± 0.07 b	0.91 ± 0.08 c	1.29 ± 0.14 b	1.90 ± 0.06 a
4-Hexen-1-Ol	7.346	1379	1391	928-92-7	0.49 ± 0.05 d	0.70 ± 0.07 c	1.77 ± 0.11 a	2.02 ± 0.10 a	1.63 ± 0.37 a	1.87 ± 0.15 a
1-Decanol	12.111	1754	1760	112-30-1	0.40 ± 0.09 b	0.35 ± 0.01 b	0.32 ± 0.02 b	1.20 ± 0.11 a	0.52 ± 0.08 b	0.31 ± 0.01 b
Nonan-1-Ol	11.233	1658	1660	143-08-8	3.70 ± 0.52 b	2.70 ± 0.21 c	2.13 ± 0.09 cd	1.56 ± 0.11 d	2.79 ± 0.37 c	4.42 ± 0.26 a
1-Heptanol	8.438	1450	1453	111-70-6	7.00 ± 0.93 b	5.07 ± 0.14 c	4.92 ± 0.30 c	3.88 ± 0.17 d	6.77 ± 0.83 b	9.79 ± 0.36 a
Menthol	14.445	1643	1644	2216-51-5	7.14 ± 1.58 bc	7.39 ± 0.80 b	6.23 ± 0.30 c	4.34 ± 0.35 d	14.70 ± 2.07 a	6.52 ± 0.19 c
	Alkenes									
(+/−)-Limonene	4.609	1196	1199	138-86-3	10.74 ± 1.68 d	33.87 ± 3.93 b	9.68 ± 0.63 d	53.97 ± 2.66 a	25.97 ± 7.33 c	8.50 ± 0.47 d
Terpinolene	5.836	1280	1281	586-62-9	7.98 ± 0.99 c	20.63 ± 1.33 a	4.53 ± 0.21 d	47.53 ± 2.50 a	16.27 ± 2.37 b	6.75 ± 0.25 c
γ-Terpinene	5.152	1234	1238	99-85-4	11.61 ± 1.48 a	9.55 ± 0.77 b	7.28 ± 0.34 c	13.97 ± 1.12 a	8.72 ± 1.66 b	3.30 ± 0.17 d
Calamenene	12.714	1833	1839	72937-55-4	11.37 ± 1.18 c	45.77 ± 4.25 a	7.72 ± 0.37 d	6.89 ± 0.51 d	62.70 ± 5.98 a	7.45 ± 0.48 d
Longicyclene	9.095	1493	1519	1137-12-8	3.77 ± 0.25 d	8.39 ± 0.79 b	10.31 ± 0.60 a	7.53 ± 0.89 bc	7.49 ± 1.23 bc	3.91 ± 0.41 d
α-Cubebene	8.516	1455	1463	31141-66-9	5.54 ± 0.68 c	14.77 ± 1.37 a	1.79 ± 0.09 d	1.45 ± 0.11 d	20.97 ± 2.16 a	1.75 ± 0.04 d
(3 E)-β-Ocimene	5.392	1250	1250	13877-91-3	11.05 ± 1.39 c	23.43 ± 1.59 b	16.37 ± 0.67 b	40.37 ± 2.36 a	15.33 ± 1.17 b	8.34 ± 3.56 c
α-Terpinene	4.348	1176	1178	99-86-5	7.83 ± 0.97 b	8.87 ± 0.94 a	5.61 ± 0.45 d	12.73 ± 0.50 a	8.35 ± 2.42 b	2.88 ± 0.04 e
β-Pinene	4.155	1160	1116	127-91-3	16.10 ± 4.69 c	26.40 ± 7.63 b	25.37 ± 0.45 b	36.87 ± 1.75 a	22.13 ± 7.76 b	12.41 ± 4.39 d
Copaene	9.714	1536	1492	3856-25-5	0.42 ± 0.06 c	1.04 ± 0.09 a	0.42 ± 0.02 c	30.13 ± 1.76 a	1.29 ± 0.13 a	14.93 ± 0.91 b
α-Corocalene	14.15	2068	2060	20129-39-9	0.25 ± 0.04 c	1.31 ± 0.13 a	0.15 ± 0.01 d	0.15 ± 0.02 d	2.09 ± 0.19 a	0.14 ± 0.01 d
α-Phellandrene	4.767	1208	1167	99-83-2	0.42 ± 0.03 b	0.49 ± 0.01 a	0.45 ± 0.01 b	0.66 ± 0.06 a	0.40 ± 0.28 b	0.27 ± 0.01 c
	Acids									
Nonanoic Acid	14.655	2166	2171	112-05-0	18.17 ± 3.49 b	24.47 ± 1.94 a	15.93 ± 1.65 c	8.79 ± 0.81 d	15.43 ± 2.67 c	19.87 ± 0.90 b
Caproic Acid	12.797	1845	1846	142-62-1	24.13 ± 3.23 c	38.57 ± 1.82 a	24.87 ± 2.05 c	16.37 ± 0.93 d	29.90 ± 4.97 b	35.70 ± 1.90 a
Geranic Acid	15.478	2339	2347	459-80-3	8.00 ± 1.39 b	7.06 ± 0.48 c	43.50 ± 2.42 a	8.28 ± 0.54 b	6.86 ± 1.35 c	5.42 ± 0.08 d
Heptanoic Acid	13.488	1952	1950	111-14-8	16.47 ± 3.30 a	14.83 ± 0.80 b	13.60 ± 0.72 c	5.37 ± 0.31 e	11.72 ± 2.02 d	15.63 ± 0.68 b
Octanoic Acid	14.101	2059	2060	124-07-2	7.67 ± 1.29 c	10.36 ± 1.23 a	5.62 ± 0.30 d	5.14 ± 0.59 d	10.03 ± 1.77 a	9.03 ± 0.52 b
Benzoic Acid	16.048	2459	2414	65-85-0	2.32 ± 0.33 d	5.25 ± 0.53 a	3.78 ± 0.43 b	3.08 ± 0.99 c	6.27 ± 1.46 a	3.64 ± 0.31 bc
2-Ethylbutyric Acid	11.673	1701	1711	32391	39.30 ± 3.95 c	59.40 ± 4.45 b	43.27 ± 4.64 c	33.77 ± 1.46 d	70.43 ± 9.90 a	41.60 ± 1.44 c
	Pyrazines									
2,3-Dimethylpyrazine	6.729	1339	1344	5910-89-4	3.46 ± 0.36 c	11.43 ± 0.72 a	5.32 ± 0.45 b	4.08 ± 0.19 c	5.30 ± 1.00 b	1.88 ± 0.15 d
2-Ethylpyrazine	6.56	1328	1337	13925-00-3	3.55 ± 0.32 d	17.83 ± 1.27 a	11.53 ± 0.90 b	5.53 ± 0.30 d	9.66 ± 1.52 c	3.14 ± 0.37 d
2,5-Dimethylpyrazine	6.364	1316	1320	123-32-0	69.60 ± 6.05 d	236.33 ± 12.50 a	184.33 ± 11.93 b	85.10 ± 4.51 d	143.33 ± 19.86 c	56.63 ± 3.55 e
2,6-Dimethylpyrazine	6.454	1322	1328	108-50-9	18.83 ± 1.82 c	99.27 ± 5.62 a	62.00 ± 4.73 b	27.53 ± 1.33 c	53.27 ± 7.05 b	18.33 ± 1.39 c
2,6-Diethylpyrazine	8.088	1428	1445	13067-27-1	0.83 ± 0.11 b	2.99 ± 0.21 a	1.31 ± 0.12 b	0.70 ± 0.03 c	1.14 ± 0.18 b	0.30 ± 0.03 d
2,3,5-Trimethylpyrazine	7.597	1396	1395	14667-55-1	18.33 ± 2.29 c	39.00 ± 2.96 a	20.40 ± 1.35 c	10.97 ± 0.59 d	16.93 ± 2.66 c	6.35 ± 0.33 e
3-Ethyl-2,5-Dimethylpyrazine	8.238	1437	1435	13360-65-1	76.70 ± 9.44 d	223.67 ± 15.04 a	128.33 ± 8.33 b	58.00 ± 2.86 e	116.67 ± 16.50 b	34.40 ± 1.65 f
2-Methylpyrazine	5.542	1260	1266	109-08-0	9.44 ± 0.67 d	58.87 ± 3.61 a	37.90 ± 3.29 b	19.83 ± 1.27 c	34.50 ± 6.24 b	13.17 ± 1.02 c
2-Ethyl-5-Methylpyrazine	7.424	1384	1383	13360-64-0	48.73 ± 28.26 c	178.00 ± 10.82 a	130.67 ± 8.74 b	63.00 ± 3.20 c	76.90 ± 37.10 c	26.77 ± 12.55 d
2,5-Diethylpyrazine	8.989	1486	1456	13238-84-1	2.41 ± 0.34 c	6.69 ± 0.57 a	3.21 ± 0.24 b	1.55 ± 0.09 d	2.71 ± 0.43 bc	0.63 ± 0.03 e
2,3-Diethyl-5-Methylpyrazine	9.319	1508	1486	18138-04-0	1.14 ± 0.18 c	2.93 ± 0.17 a	1.22 ± 0.08 c	0.75 ± 0.06 d	1.11 ± 0.18 c	0.27 ± 0.02 e
Acetylpyrazine	10.848	1621	1631	22047-25-2	0.41 ± 0.05 c	1.37 ± 0.10 a	0.81 ± 0.04 b	0.37 ± 0.02 c	0.79 ± 0.10 b	0.47 ± 0.02 c
3,5-Diethyl-2-Methylpyrazine	9.273	1505	1508	18138-05-1	3.65 ± 0.55 b	6.57 ± 0.56 a	2.71 ± 0.21 c	1.74 ± 0.09 d	2.52 ± 0.39 c	0.62 ± 0.02 e
	Furans									
2-Ethylfuran	2.152	969	951	3208-16-0	2.03 ± 0.23 c	2.52 ± 0.17 b	2.22 ± 0.21 c	1.87 ± 0.11 c	2.56 ± 0.42 b	3.26 ± 0.22 a
2-Acetylfuran	9.205	1500	1499	1192-62-7	18.27 ± 1.67 d	98.30 ± 5.57 a	60.03 ± 4.20 b	40.27 ± 2.57 c	60.10 ± 8.15 b	20.37 ± 1.47 d
2-Pentylfuran	5.098	1230	1232	3777-69-3	51.87 ± 8.04 c	58.57 ± 2.91 b	43.63 ± 2.77 d	35.07 ± 1.69 e	48.33 ± 5.25 c	66.10 ± 5.21 a
2-(2-Pentenyl) Furan	6.142	1301	1282	70424-14-5	1.00 ± 0.12 d	1.83 ± 0.13 a	1.18 ± 0.10 c	1.62 ± 0.10 b	1.55 ± 0.23 b	2.07 ± 0.15 a
2-Methylfuran	1.848	937	870	534-22-5	0.74 ± 0.06 c	1.16 ± 0.08 b	1.64 ± 0.14 a	0.77 ± 0.03 c	1.30 ± 0.23 b	1.34 ± 0.08 b
	Pyrroles									
1-Furfurylpyrrole	12.684	1829	1824	1438-94-4	0.37 ± 0.04 c	2.63 ± 0.21 a	1.35 ± 0.14 b	0.58 ± 0.05 c	0.64 ± 0.12 c	0.04 ± 0.03 d
2-Acetylpyrrole	13.606	1971	1973	1072-83-9	16.03 ± 2.18 d	56.20 ± 3.26 a	39.67 ± 1.38 b	22.17 ± 1.38 c	43.43 ± 5.17 b	9.23 ± 0.32 e
	Miscellaneous									
Vanillin	16.716	2577	2569	121-33-5	5.89 ± 1.08 c	18.77 ± 0.72 a	9.84 ± 0.73 b	11.90 ± 0.36 ab	11.43 ± 1.01 b	12.80 ± 0.30 a
1,1,6-Trimethyl-1,2-Dihydronaphthalene	12.016	1742	1737	30364-38-6	6.97 ± 0.94 b	11.03 ± 0.83 a	6.25 ± 0.47 c	7.13 ± 0.49 b	6.23 ± 0.90 c	1.32 ± 0.07 d
Toluene	2.8	1038	1042	108-88-3	7.39 ± 0.53 c	16.60 ± 1.51 a	9.25 ± 0.65 b	9.80 ± 0.52 b	10.57 ± 2.06 b	4.46 ± 0.42 d
Naphthalene	11.971	1737	1745	91-20-3	11.33 ± 1.25 bc	13.60 ± 2.25 ab	12.67 ± 1.00 b	15.40 ± 1.40 a	13.73 ± 1.55 ab	8.51 ± 0.68 c
Ethylbenzene	3.693	1124	1129	100-41-4	0.95 ± 0.07 b	1.31 ± 0.24 a	1.23 ± 0.03 a	0.99 ± 0.02 b	1.38 ± 0.22 a	0.77 ± 0.07 c
2,4-Di-Tert-Butylphenol	15.327	2306	2321	96-76-4	119.87 ± 19.69 a	108.67 ± 8.33 b	102.30 ± 4.10 b	139.67 ± 15.18 a	81.97 ± 5.66 c	59.70 ± 1.44 d
Tridecane	6.613	1332	1300	629-50-5	0.60 ± 0.07 c	0.85 ± 0.06 a	0.75 ± 0.04 b	0.64 ± 0.05 c	1.20 ± 0.35 a	0.58 ± 0.03 c
Tetradecane	8.255	1439	1400	629-59-4	2.91 ± 0.33 c	5.11 ± 0.39 a	4.73 ± 0.37 a	4.95 ± 0.24 a	4.38 ± 0.51 b	2.94 ± 0.09 c
P-Cymene	5.665	1269	1272	99-87-6	8.26 ± 0.89 c	11.23 ± 0.96 b	7.27 ± 0.36 d	15.47 ± 1.01 a	8.39 ± 1.42 c	3.61 ± 0.25 e
Fluorene	15.568	2359	2337	86-73-7	0.76 ± 0.14 c	1.37 ± 0.15 a	0.89 ± 0.05 b	0.62 ± 0.04 c	1.24 ± 0.13 a	0.81 ± 0.03 b
Hexadecane	10.574	1596	1600	544-76-3	1.61 ± 0.21 c	2.65 ± 0.23 a	2.52 ± 0.16 a	1.95 ± 0.16 b	3.14 ± 0.31 a	1.76 ± 0.09 c
O-Xylene	4.416	1181	1183	95-47-6	3.04 ± 0.29 c	4.51 ± 0.66 a	3.61 ± 0.13 b	2.49 ± 0.12 d	4.06 ± 0.39 ab	2.27 ± 0.23 d
Indole	16.054	2460	2469	120-72-9	9.96 ± 1.46 c	26.23 ± 2.05 a	17.77 ± 0.59 b	10.32 ± 0.73 c	10.97 ± 1.65 c	17.47 ± 0.42 b
Pentadecane	9.203	1500	1500	629-62-9	1.47 ± 0.27 c	1.88 ± 0.21 b	1.93 ± 0.22 b	1.64 ± 0.06 bc	2.19 ± 0.16 a	1.15 ± 0.04 d
Skatole	16.3	2508	2515	83-34-1	0.90 ± 0.15 c	1.34 ± 0.11 a	0.87 ± 0.05 c	1.27 ± 0.08 ab	0.71 ± 0.09 d	0.43 ± 0.01 e
Eugenol	14.683	2171	2169	97-53-0	0.26 ± 0.05 d	0.94 ± 0.07 a	0.32 ± 0.01 cd	0.32 ± 0.02 cd	0.63 ± 0.08 b	1.40 ± 0.02 a
Coumarin	16.131	2476	2465	91-64-5	2.53 ± 0.33 d	38.13 ± 2.56 a	7.90 ± 0.22 c	3.50 ± 0.17 d	8.50 ± 0.69 c	13.90 ± 0.44 b

Data represent mean ± SD, and means with the same letter are not significantly different from each other at *p* < 0.05.

**Table 2:** Aroma compounds with an rOAV > 1 in 6 different tea cultivars.

Metabolite	CAS ID	Threshold (μg/L or μg/kg)	HJC1	CC4H	GZSC	QM601	SLX	SMCY
Nonanal	124-19-6	1.1	25.67 ± 4.42	29.58 ± 2.58	18.48 ± 0.73	12.06 ± 0.14	18.12 ± 2.09	25.15 ± 0.82
Hexanal	66-25-1	4.5	1.21 ± 0.18	1.33 ± 0.09	1.13 ± 0.06	0.80 ± 0.05	1.10 ± 0.06	1.38 ± 0.08
β-Cyclocitral	432-25-7	3	2.49 ± 0.30	3.73 ± 0.44	3.92 ± 0.27	3.42 ± 0.21	4.02 ± 0.66	3.26 ± 0.12
Benzaldehyde	100-52-7	0.024	3475.00 ± 421.33	3577.78 ± 187.33	2804.17 ± 147.96	6986.11 ± 479.32	3565.28 ± 497.94	4512.50 ± 309.49
2-Methylbutyraldehyde	96-17-3	1.5	11.42 ± 1.12	6.75 ± 0.27	7.28 ± 0.68	31.24 ± 2.01	12.16 ± 2.04	4.20 ± 0.33
Octanal	124-13-0	0.8	5.89 ± 0.97	6.85 ± 0.60	3.78 ± 0.27	2.60 ± 0.04	4.22 ± 0.62	6.29 ± 0.33
Heptanal	111-71-7	2.8	4.20 ± 0.71	3.48 ± 0.32	3.26 ± 0.18	1.51 ± 0.02	2.90 ± 0.36	3.11 ± 0.23
Tetradecanal	124-25-4	67	1.16 ± 0.14	0.35 ± 0.03	0.41 ± 0.02	0.15 ± 0.00	1.64 ± 0.12	0.44 ± 0.00
Safranal	116-26-7	3	3.28 ± 0.38	3.93 ± 0.40	3.90 ± 0.23	3.09 ± 0.24	3.67 ± 0.51	3.64 ± 0.18
Isovaleraldehyde	590-86-3	0.5	11.12 ± 1.26	2.22 ± 0.08	2.51 ± 0.20	10.22 ± 0.62	6.81 ± 1.15	5.17 ± 0.39
Decanal	112-31-2	3	0.81 ± 0.21	0.87 ± 0.01	0.70 ± 0.04	0.56 ± 0.02	1.01 ± 0.33	0.70 ± 0.17
1-Octen-3-One	4312-99-6	0.003	584.44 ± 84.74	840.00 ± 87.43	654.44 ± 55.21	531.11 ± 59.66	947.78 ± 110.02	998.89 ± 57.48
Jasmone	488-10-8	1.9	1.82 ± 0.27	10.63 ± 0.92	3.57 ± 0.11	6.14 ± 0.51	7.91 ± 0.97	16.23 ± 0.21
β-Ionone	79-77-6	0.021	1315.87 ± 174.60	963.49 ± 116.87	931.75 ± 48.64	836.51 ± 9.91	1109.52 ± 127.86	963.49 ± 9.91
2,3-Octanedione	585-25-1	12	2.29 ± 0.23	2.61 ± 0.17	1.89 ± 0.35	2.25 ± 0.11	3.25 ± 0.64	2.29 ± 0.14
Damascenone	23726-93-4	0.006	53.17 ± 8.49	92.72 ± 5.59	103.06 ± 4.10	51.67 ± 3.18	111.56 ± 13.68	31.67 ± 1.17
(E)-β-Damascone	23726-91-2	0.001	616.33 ± 113.46	995.33 ± 133.81	2245.00 ± 3399.39	4166.67 ± 259.29	1266.67 ± 127.02	5253.33 ± 122.20
(E,E)-3,5-Octadien-2-One	38284-27-4	0.1	33.83 ± 3.60	86.10 ± 6.20	60.37 ± 2.06	79.67 ± 4.26	61.37 ± 8.02	110.33 ± 7.02
Methyl Salicylate	119-36-8	40	0.75 ± 0.10	1.55 ± 0.17	2.34 ± 0.13	1.75 ± 0.12	2.80 ± 0.36	1.64 ± 0.05
Linalyl Acetate	115-95-7	0.1109	4.02 ± 0.32	62.43 ± 5.32	12.71 ± 0.87	33.54 ± 2.72	23.29 ± 2.59	21.67 ± 1.01
Methyl Nonanoate	1731-84-6	0.04	1.97 ± 0.22	4.27 ± 0.23	3.46 ± 0.22	4.63 ± 0.08	3.44 ± 0.41	9.64 ± 0.49
Phenylethyl Alcohol	20-12-8	564.23	1.21 ± 0.09	0.75 ± 0.03	0.87 ± 0.02	0.79 ± 0.03	0.67 ± 0.06	1.36 ± 0.02
Geraniol	106-24-1	6.6	15.94 ± 2.18	9.24 ± 0.80	27.17 ± 1.22	10.09 ± 0.71	10.19 ± 1.28	13.35 ± 0.38
Linalool	78-70-6	6	12.11 ± 1.39	18.83 ± 1.04	7.04 ± 0.33	43.56 ± 1.87	24.00 ± 3.25	24.56 ± 1.21
1-Octen-3-Ol	3391-86-4	1.5	13.13 ± 1.63	19.40 ± 2.31	15.56 ± 1.04	12.87 ± 0.66	18.62 ± 2.90	17.53 ± 1.01
(3E)-β-Ocimene	13877-91-3	0.02	552.50 ± 69.60	1171.67 ± 79.43	818.33 ± 33.29	2018.33 ± 118.15	766.67 ± 58.59	417.17 ± 178.18
α-Phellandrene	99-83-2	0.04	10.61 ± 0.73	12.28 ± 0.28	11.23 ± 0.37	16.57 ± 1.61	9.95 ± 7.11	6.72 ± 0.32
3-Ethyl-2,5-Dimethylpyrazine	13360-65-1	25	3.07 ± 0.38	8.95 ± 0.60	5.13 ± 0.33	2.32 ± 0.11	4.67 ± 0.66	1.38 ± 0.07
2-Ethyl-5-Methylpyrazine	13360-64-0	16	3.05 ± 1.77	11.13 ± 0.68	8.17 ± 0.55	3.94 ± 0.20	4.81 ± 2.32	1.67 ± 0.78
2,5-Diethylpyrazine	13238-84-1	0.02	120.33 ± 17.22	334.67 ± 28.25	160.50 ± 12.13	77.67 ± 4.54	135.50 ± 21.50	31.28 ± 1.42
2,3-Diethyl-5-Methylpyrazine	18138-04-0	0.031	36.76 ± 5.91	94.62 ± 5.49	39.35 ± 2.64	24.13 ± 1.87	35.86 ± 5.90	8.68 ± 0.50
3,5-Diethyl-2-Methylpyrazine	18138-05-1	0.26	14.03 ± 2.13	25.27 ± 2.15	10.44 ± 0.81	6.68 ± 0.36	9.71 ± 1.49	2.38 ± 0.09
2-Pentylfuran	3777-69-3	5.8	8.94 ± 1.39	10.10 ± 0.50	7.52 ± 0.48	6.05 ± 0.29	8.33 ± 0.91	11.40 ± 0.90
P-Cymene	99-87-6	11.4	0.72 ± 0.08	0.99 ± 0.08	0.64 ± 0.03	1.36 ± 0.09	0.74 ± 0.12	0.32 ± 0.02
Indole	120-72-9	11	0.91 ± 0.13	2.38 ± 0.19	1.62 ± 0.05	0.94 ± 0.07	1.00 ± 0.15	1.59 ± 0.04
Coumarin	91-64-5	11	0.23 ± 0.03	3.47 ± 0.23	0.72 ± 0.02	0.32 ± 0.02	0.77 ± 0.06	1.26 ± 0.04

## Data Availability

The original contributions presented in this study are included in the article. Further inquiries can be directed to the corresponding author.

## Abbreviations

CC4H	Chuancha 4
SLX	Shilixiang
SMCY	Siming Cuiya
HJC1	Huangjincha 1
QM601	Qianmei 601
GZSC	Guizhou Zise Tea
HS-SPME	headspace solid-phase microextraction
GC/MS	gas chromatography-mass spectrometry
DVB/CWR/PDMS	divinylbenzene/carbon wide range/polydimethylsiloxane
rOAV	the relative odor activity value
OPLS-DA	Orthogonal partial least squares discriminant analysis

## Appendix A

**Table A1:** Sources of aroma descriptions and thresholds for key volatile compounds.

Metabolite	Odor	CAS ID	Threshold (μg/L or μg/kg)	References
Nonanal	waxy; fatty; citrus; rose; fat; orange peel; grapefruit; green; fish; fresh; orris; aldehydic; lime	124-19-6	1.1	[57]
Hexanal	fatty; leafy; fat; sweaty; green; tallow; fresh; grass; aldehydic; fruity	66-25-1	4.5	[58]
β-Cyclocitral	rose oxide; tobacco; sweet; saffron; tropical; minty; clean; herbal; damascone; mint; fruity	432-25-7	3	[59]
Benzaldehyde	cherry; vanilla; almond; fruity; sweet; caramel; sharp; strong; bitter; bitter almond	100-52-7	0.024	[60]
2-Methylbutyraldehyde	coffee; malty; almond; musty; cocoa; nutty	96-17-3	1.5	[61]
Octanal	waxy; lemon; fatty; citrus; fat; orange peel; green; aldehydic; soapy	124-13-0	0.8	[59]
Heptanal	fatty; citrus; wine-lee; fat; rancid; ozone; green; herbal; fresh; aldehydic	111-71-7	2.8	[59]
Tetradecanal	dry; musky; flower; fatty; waxy; incense; wax; citrus peel	124-25-4	67	[62]
Safranal	phenolic; spicy; tobacco; sweet; metallic; herbal; fresh; rosemary	116-26-7	3	[58]
Isovaleraldehyde	fatty; peach; malty; chocolate; sour; aldehydic	590-86-3	0.5	[61]
Decanal	waxy; citrus; sweet; orange peel; tallow; aldehydic; soapy	112-31-2	3	[59]
1-Octen-3-One	mushroom; earthy; metallic; musty; dirty; herbal	4312-99-6	0.003	[63]
Jasmone	spicy; jasmine; celery; herbal; woody; jasmine	488-10-8	1.9	[59]
β-Ionone	dry; flower; powdery; jam; seaweed; violet; orange; woody; orris; raspberry	79-77-6	0.021	[61]
2,3-Octanedione	cooked; buttery; broccoli; dill	585-25-1	12	[62]
Damascenone	apple; tobacco; rose; smoke; honey; sweet; apple. rose	23726-93-4	0.006	[61]
(E)- β-Damascone	apple; tobacco; rose; plum; honey; blackcurrant; berry; fruity	23726-91-2	0.001	[40]
(E, E)-3,5-Octadien-2-One	grassy; mushroom; fatty; fruity; green	38284-27-4	0.1	[64]
Methyl Salicylate	wintergreen; minty; peppermint	119-36-8	40	[59]
Linalyl Acetate	citrus; sweet; lavender; fruity; green; bergamot; woody	115-95-7	0.1109	[65]
Methyl Nonanoate	waxy; coconut; sweet; pear; tropical; fruity; wine	1731-84-6	0.04	[59]
Phenylethyl Alcohol	lilac; rose flower; rose water; honey; rose; rose dried; bitter; spice	20-12-8	564.23	[59]
Geraniol	waxy; citrus; rose; geranium; sweet; fruity	106-24-1	6.6	[59]
Linalool	flower; lemon; citrus; sweet; lavender; green; orange; woody; blueberry	78-70-6	6	[59]
1-Octen-3-Ol	fungal; mushroom; earthy; raw; green; fish; chicken; oily	3391-86-4	1.5	[59]
(3E)-β-Ocimene	woody; citrus; herbal; tropical; green; sweet; terpene	13877-91-3	0.02	[66]
α-Phellandrene	citrus; peppery; green; turpentine; woody; herbal; terpene; minty; spice	99-83-2	0.04	[67]
3-Ethyl-2,5-Dimethylpyrazine	cocoa; nutty; potato; roast; roasted	13360-65-1	25	[61]
2-Ethyl-5-Methylpyrazine	sweet; bean; fruity; coffee	13360-64-0	16	[61]
2,5-Diethylpyrazine	-	13238-84-1	0.02	[61]
2,3-Diethyl-5-Methylpyrazine	vegetable; wine; earthy; meat; potato; citrus; fatty; roast; musty; herbal; nutty; spicy; green; hazelnut; woody; fruity; meaty	18138-04-0	0.031	[61]
3,5-Diethyl-2-Methylpyrazine	vegetable; nutty; roast; meaty	18138-05-1	0.26	[61]
2-Pentylfuran	buttery; earthy; metallic; green bean; green; vegetable; fruity; beany	3777-69-3	5.8	[61]
P-Cymene	solvent; citrus; gasoline; terpene; woody; fresh; spice	99-87-6	11.4	[58]
Indole	fecal; moth ball; fish; jasmine; honey; animal; naphthelene; mothball; burnt	120-72-9	11	[59]
Coumarin	bitter; sweet; green; new mown hay	91-64-5	11	[61]
